# Imperforate Anus with False Opening: Anus Vulvalis

**Published:** 1895-09

**Authors:** J. T. Duncan

**Affiliations:** Professor of Anatomy, Ontario Veterinary College


### IMPERFORATE ANUS WITH FALSE OPENING: ANUS VULVALIS.

BY J. T. DUNCAN, M.D., V.S.,
PROFESSOR OF ANATOMY, ONTARIO VETERINARY COLLEGE.

THIS interesting abnormality, viz., the opening of the intestine into the vulva, and the passage of the feces through the cavity of the vulva, is a rare one in the full-grown animal, not, however, so uncommon in the young.

Fleming,[1] under the heading of "imperforation of the anus," thus speaks of the condition: "In young female animals in which the rectum (anus) is absent it often happens that the intestine opens into the vagina, and the feces are expelled through that passage."

In discussing or reporting cases of this peculiarity it will be well to come to some agreement as to terms, there being a good deal of looseness at present in this matter. First, in reference to the name to be adopted. From among the names used I have selected two for the head of this article, but the first is cumbersome and indefinite, the latter short and explicit. I shall therefore take the latter as the preferable term or name.

What do we, as veterinarians, understand by the term vulva? The vulva is a passage extending from the hymen anteriorly to the labia posteriorly, being (in the mare) about five inches in length. The vagina, on the other hand, is a passage extending from the os uteri to the hymen. We speak then of anus vulvalis because the intestine opens into the cavity of the vulva. In those extremely rare cases in which the intestine does open into the vagina (*i. e.*, when the opening is more than five inches from the labia) the term anus vaginalis would be allowable.

[1] Veterinary Obstetrics, by Dr. G. Fleming, Am. ed., 1881.

Cases of anus vulvalis are comparatively common in the young, but extremely rare in the full-grown animal. The reason for this is twofold; First, many young animals, by an operation, have communication established between the intestine and the anus, the false opening into the vulva then closing up. Secondly, many young animals, not operated upon, perish in early life. A third reason might be added, viz. some of the operations are not successful, resulting in death.

The condition, rare though it be, should be more clearly understood than it is, and if all the members of the veterinary profession were to publish, in the various veterinary journals, cases coming under their notice, with the results of operations performed, the profession would soon be in possession of valuable data in regard to it. It may be of interest to glance, for a moment, at the subject in connection with the human species. Medical men have placed many cases of this abnormality on record; the vast majority, however, being in infants. The opening into the vulva, in some of these cases, was a mere pinhole in point of size; in others it was double; and in still others the intestine opened into the anus in a normal manner, but it also opened into the vulva, the feces escaping, of course, in both directions. In some no sphincter could be discovered. In such cases the patient is unable to control the lower bowel; in other words, there is incontinence of feces. Most of those infantile cases are successfully operated upon, but some are allowed to go without anything being done. If the vulval opening be large and controlled by a sufficient sphincter, the adult condition may be attained in good health.

The manner in which this abnormality is produced is essentially the same in the human being and in the lower animal. A brief reference to the development of the intestine will help to the understanding of it. Speaking now of the lower animals, the intestine in early fœtal life is a tube, closed at both extremities. There is at first no appearance of a mouth, nor yet of an anus; but a depression appears at each extremity of the embryo, the one in the position of the mouth, the other in that of the anus. Both of these cavities or pouches are lined by common integument, and both are separated from the intestinal tube by a septum more or less thick. By the development of the intestinal tube and the deepening of the cavities just spoken of, the septa are caused to disappear, and the tube is now open from end to end. The cavity at the posterior extremity has opening into it not only the intestine, but the generative and urinary passages. It is, therefore, a chamber common to all three systems, and may be spoken of as a cloaca.

Gradually the cloacca is subdivided by the growth of a transverse plate or band of integumental tissue. Above this plate lies the intestine, opening at the anus; below, the cavity of the vulva. The plate, if complete, entirely separates one from the other. If, now, invagination of the integument has not taken place above the transverse plate (or not to a sufficient extent), and the plate itself is not complete, we can very readily understand how the condition is that of anus vulvalis.

